# Correlation between cortical lesions and cognitive impairment in multiple sclerosis

**DOI:** 10.1002/brb3.955

**Published:** 2018-04-21

**Authors:** Erica Curti, Stefania Graziuso, Elena Tsantes, Girolamo Crisi, Franco Granella

**Affiliations:** ^1^ Neurosciences Unit Department of Medicine and Surgery (DMEC) University of Parma Parma Italy; ^2^ Neuroradiology Unit Department of Diagnostic Parma University Hospital Parma Italy

**Keywords:** cognitive impairment, cortical lesions, double inversion recovery, multiple sclerosis

## Abstract

**Objectives:**

Gray matter (GM) damage is well known as a fundamental aspect of multiple sclerosis (MS). Above all, cortical lesions (CLs) burden, detectable at MRI with double inversion recovery (DIR) sequences, has been demonstrated to correlate with cognitive impairment (CI). The aim of this study was to investigate the role of CLs number in predicting CI in a cohort of patients with MS in a clinical practice setting.

**Materials and methods:**

Thirty consecutive patients with MS presenting CLs (CL+) at high‐field (3.0 T) MRI 3D‐DIR sequences and an even group of MS patients without CLs (CL‐) as a control, were investigated with the Rao Brief Repeatable Battery of Neuropsychological Tests (BRB), Version A. Total and lobar CLs number were computed in CL+ patients.

**Results:**

Among the sixty patients with MS enrolled, forty‐seven (78.3%) had a relapsing‐remitting course, while thirteen (21.7%) a progressive one, eleven secondary progressive, and two primary progressive. Compared to CL−, CL+ patients had a greater proportion of progressive forms (*p* = .03). The most affected region was the frontal lobe (73.3% of patients), followed by temporal and parietal ones (both 60.0%). Multivariate (logistic regression) analysis revealed a significant correlation between total CLs number and the presence of mild cognitive impairment defined as pathologic score in at least one BRB test (*p* = .04); it was also correlated with deficit at PASAT 3 (*p* = .05) and Stroop Test (*p* = .02).

**Conclusions:**

We confirmed CLs number, evaluated with a technique quite commonly available in clinical practice, as a predictive factor of CI in patients with MS, in order to improve the diagnosis and management of CI and monitor potential neuroprotective effects of therapies.

## 1 INTRODUCTION

Multiple sclerosis (MS) is the most common immune‐mediated, chronic, inflammatory disease of the central nervous system, typically affecting the white matter (WM) in the form of multifocal areas of demyelination, as historically known. Nevertheless, in recent years, several neuropathological and imaging studies have demonstrated also the involvement of the cortical and deep gray matter (GM), in the form of an inflammatory process, both focal and diffuse, in addition to a neurodegenerative one leading to cerebral atrophy (Lucchinetti et al., 2011; Pirko, Lucchinetti, Sriram, & Bakshi, 2007; Popescu & Lucchinetti, 2012). Moreover, there are many evidences supporting the correlation between GM damage, measured either as cortical lesion (CL) load or as cortical atrophy, and both physical and cognitive impairment (CI) (Calabrese et al., 2007, 2009, 2009; Damasceno, Damasceno, & Cendes, 2015; De Stefano et al., 2003; Geurts, Calabrese, Fisher, & Rudick, 2012; Mike et al., 2011; Nelson et al., 2011; Papadopoulou et al., 2013; Roosendaal et al., 2009).

Because conventional MRI techniques cannot easily reveal GM lesions, more specific sequences have been introduced, such as double inversion recovery (DIR), which selectively suppresses signal from WM and cerebrospinal fluid (CSF), improving the detection of CLs (Geurts et al., 2005; Turetschek et al., 1998). International guidelines have been proposed to facilitate detection of CLs (Geurts et al., 2011). Moreover, a three‐dimensional version (3D‐DIR) allows for a shorter acquisition time and a reduction in artefacts (Roosendaal et al., 2009). With the application of these techniques, several studies have demonstrated that CLs are a typical finding in all phenotypes and stages of MS, including clinically isolated syndrome (CIS) (Calabrese et al., 2007, 2008; Geurts et al., 2005) and radiologically isolated syndrome (Calabrese & Gallo, 2009; Giorgio et al., 2011), although they are more represented in the progressive forms and with longer disease duration (Calabrese et al., 2015; Kutzelnigg et al., 2005; Mike et al., 2011; Roosendaal et al., 2009).

Cognitive impairment is an important component of MS‐related disability at both the earlier and later stages of the disease, affecting from 40% to 70% of patients with MS. Several cognitive domains, such as long‐term memory, attention and concentration, executive functioning, and information processing speed (IPS), may be involved (Amato, Zipoli, & Portaccio, 2006; Chiaravalloti & DeLuca, 2008; Jongen, Ter Horst, & Brands, 2012; Lovera & Kovner, 2012). In recent years, some MRI studies using DIR sequences have demonstrated a significant correlation between CLs burden, mainly measured as CLs volume, and cognitive dysfunction in patients with MS (Calabrese et al., 2007, 2009; Damasceno et al., 2015; Mike et al., 2011; Nelson et al., 2011; Papadopoulou et al., 2013; Roosendaal et al., 2009).

Although there is also some evidence of a correlation between regional GM and WM damage measures and performances at neuropsychological tests (Morgen et al., 2006; Nocentini et al., 2014; Sperling et al., 2001), in our knowledge, there are only sporadic reports of a correlation between regional CLs number and impairment within specific cognitive domains (Roosendaal et al., 2009).

With this study, conducted with high‐field 3.0 Tesla (T) MRI 3D‐DIR sequence, a technique nowadays quite available in the clinical practice setting in Europe, we aimed to investigate the role of CLs load, measured as CLs number, in predicting CI in a cohort of patients affected by relapsing‐remitting (RR) and progressive MS. We also analyzed the regional distribution of CLs in the various cerebral lobes and their relative contribution to CI.

## 2 MATERIALS AND METHODS

### 2.1 Patients

We enrolled a group of thirty consecutive patients with MS diagnosis according to revised 2010 McDonald criteria, referred to MS Centre of Parma between October 2014 and January 2015 and presenting CLs (CL+) at a brain MRI scan with DIR sequences. An even group of consecutive MS patients without CLs (CL−) was enrolled as a control. All individuals were investigated for MS‐related CI with specific neuropsychological tests. Depression was evaluated by the Beck Depression Inventory (BDI) (Hautzinger, 1991), with regard to the well‐known influence of mood disorders on cognitive performance. Patients with severe depression (BDI > 31) were excluded from the study. The study protocol was approved by the “Ethics Committee Province of Parma,” and all subjects gave written informed consent prior to participation.

### 2.2 Image acquisition

All MR images were obtained with a 3.0 T MR scanner (Discovery MR750; GE Healthcare, Milwaukee, WI) equipped with an 8‐channel phased‐array head coil. We performed 3D fast spin echo (Cube) DIR T2‐weighted pulse sequence in order to obtain optimal GM‐WM contrast for optimal visualization of cortical MS lesions. This technique is based on 3D‐FSE‐XETA (eXtended Echo Train Acquisition), an advancement of 3D‐FSE in which refocusing flip angle modulation and 2D acceleration enable volumetric coverage with high in‐ and through‐plane resolution within clinical scan times of 3–6 min. Cube DIR sequences were obtained using two adiabatic inversion recovery RF pulses to null the signal from CSF and WM. The TI values for nulling the CSF were set at 3,000–5,000 ms (TIcsf) and WM (TIwm) at 750–800 ms. The Cube DIR sequence parameters were as follows: TE/TR 85/6,900 ms, receiver bandwidth 62.50 kHz, FOV 24 × 24 cm, imaging matrix 256 × 256, section thickness 1.6 mm, 116 sections, NEX 2.0 with parallel imaging acceleration factor 2.0. Intrinsic low SNR was exceeded increasing NEX.

### 2.3 Image analysis

All images were examined by consensus by two experienced observers (S.G. and G.C.) who were blinded to the patients' identity and clinical features. With regard to the scoring of CLs on DIR images, both examiners followed the recommendations proposed by Geurts et al. (2011), with particular attention to avoid artefacts. For each patient, total, frontal, temporal, parietal, occipital CLs number, and total T2/FLAIR lesions number were assessed. The different lobes were defined based on anatomic atlas. The scoring was mainly focused on leukocortical and intracortical CLs, because of poor sensitivity of DIR sequences in the detection of subpial lesions (Geurts et al., 2011, 2012).

### 2.4 Neuropsychological evaluation

Neuropsychological assessment using the Rao Brief Repeatable Battery of Neuropsychological Tests (BRB), Version A (Amato et al., 2006) plus Stroop Test (ST) was performed within 30 days from MRI scan by a specifically trained examiner blinded to both clinical and radiological results. A 100‐item version of the ST was applied, considering as performance score the time to name 50 items in the “color‐word condition” (Amato et al., 2006). The cognitive domains investigated by these tests include verbal immediate and delayed recall memory with the Selective Reminding Test (SRT) and Selective Reminding Test–Delayed Recall (SRT‐D), spatial immediate and delayed recall memory with 10/36 Spatial Recall Test and Spatial Recall Test–Delayed (SPART and SPART‐D), sustained attention, concentration, IPS with Paced Auditory Serial Addition Test at 3 and 2 s (PASAT 3 and PASAT 2) and Symbol Digit Modalities Test (SDMT), and verbal fluency on semantic stimulus with Word List Generation (WLG). The ST evaluates attention and some aspects of executive functions, such as “the ability to elaborate relevant and irrelevant dimensions in parallel and to inhibit an automatic response while performing a task based on conflicting stimuli” (Amato et al., 2006).

Raw score at each test was corrected considering also age, gender, and education, according to normative Italian data (Amato et al., 2006). Tests with scores 2 SDs below the mean normative values were considered pathological. Patients with at least one altered test were considered affected by mild cognitive impairment (MCI), while patients with at least two altered tests were considered affected by definite cognitive impairment (DCI).

### 2.5 Statistical analysis

Chi‐square test, Student's *t* test, and median test were used to compare demographic and clinical features between the two groups of patients, with and without CLs at DIR sequences, for categorical and continuous variables, respectively.

To investigate the relationship between MRI and neuropsychological variables, univariate correlations between continuous variables were assessed using the Pearson correlation coefficient.

A multivariate analysis (logistic regression) was performed to determine the relative contribution of demographic (age and gender), clinical (EDSS, disease duration and phenotype), and MRI (total, frontal, temporal, and parietal CLs number and total T2/FLAIR lesions number) variables in predicting the presence of DCI, MCI, and pathologic performance in single BRB tests. We also applied the same multivariate analysis in a subgroup including only patients affected by the RR phenotype.

All statistical analyses were performed using SPSS version 22 (IBM Corp.) statistical package.

## 3 RESULTS

The total of sixty patients included in the study had the following demographic and clinical features: 42 (70.0%) were women and 18 (30.0%) men; mean age was 39.5 ± 11.13 years (range 18–67 years); mean age at MS onset was 30.7 ± 9.33 years (range 11–54 years); mean disease duration was 101.2 ± 86.87 months (range 2–364 months); median EDSS was 2.0 (range: 1.0–6.5). With regard to disease phenotypes, 47 patients (78.3%) had a RR course, while 13 (21.7%) had a progressive course, eleven secondary progressive (SP), and two primary progressive (PP). Compared to CL− patients, CL+ patients had a greater proportion of progressive forms (*p* = .03) (Table 1).

**Table 1:** Demographic and clinical features of patients with (CL+) and without (CL−) cortical lesions

	CL+ (*n* = 30)	CL− (*n *=* *30)	P
Mean age (years ± *SD*)	39.5 ± 12.61	39.6 ± 9.64	0.98
Gender (% female)	76.7	63.3	0.26
Mean age at MS onset (years ± *SD*)	29.5 ± 10.70	31.9 ± 7.71	0.32
Disease duration (month)	108.1 ± 74.94	94.3 ± 98.17	0.54
Median EDSS	2.5	2.0	0.19
MS type (%)			
RR	66.7	90.0	0.03
P	33.3	10.0	

CL, cortical lesion; *SD*, standard deviation; MS, multiple sclerosis; EDSS, Expanded Disability Status Scale; RR, relapsing‐remitting; P, progressive.

### 3.1 Cortical lesions

Among the thirty patients CL+, we found a total of 172 CLs, 83 frontal (48.3%), 57 temporal (33.1%), 31 parietal (18.0%), and a single occipital lesion (0.6%).

**Figure 1:**
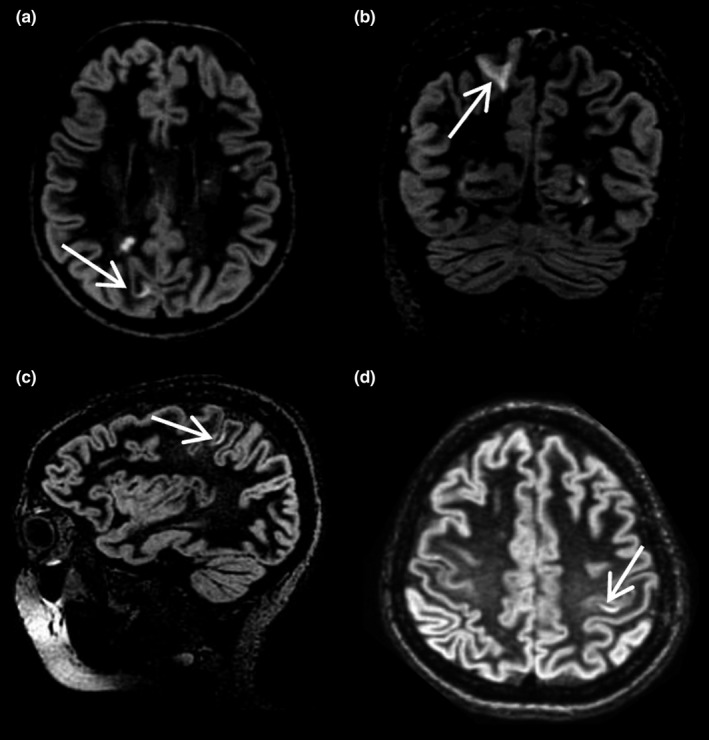
(a‐d) Examples of cortical lesions (white arrows) detected with 3D‐DIR in axial (a and d), coronal (b), and sagittal (c) scans in our population

In more detail, with regard to the regional pattern of distribution of CLs in CL+ patients, we found that the most affected area was the frontal lobe (22 patients, 73.3%), with a mean burden per patient of 3.8 ± 3.85 lesions (range 1–15), followed by temporal (18 patients, 60.0%), with a mean burden of 3.2 ± 2.90 lesions (range 1–10) per patient, and parietal lobe (18 patients, 60.0%) with a mean burden of 1.7 ± 1.32 lesions (range 1–6) per patient. The occipital lobe was spared in all patients but one. In Figure 1, there are some examples of DIR CLs images in our population.

### 3.2 T2/FLAIR lesions

Considering all our sample, we found a mean value of T2/FLAIR hyperintense lesions of 22.7 ± 16.42 (range 2–63) per patient. In the CL+ group, we detected a mean value of 29.2 ± 16.30 (range 5–63) T2/FLAIR lesions per patient, while of 16.2 ± 13.99 (range 2–50) in the other group (*p* = .002). Total T2/FLAIR lesions number was higher in the progressive group, with a mean value of 32.8 ± 18.62 vs 19.9 ± 14.78 in the RRMS group (*p* = .01).

### 3.3 Cognitive impairment

We found DCI, determined by failure in ≥2 BRB tests with scores 2 *SD* below the mean value score, in 11 patients (18.3%), while MCI, defined by failure in ≥1 BRB test, was present in 24 patients (40.0%).

Analyzing the frequency of CI in the two groups of patients CL+ and CL−, we found a greater proportion of patients with both DCI and MCI in the CL+ group, even though they did not reach the statistical significance (23.3% of CL+ vs 13.3% of CL− patients with DCI, *p* = .51; 43.3% of CL+ vs 36.7% of CL− patients with MCI, *p* = .60).

Analyzing the relationship between performance at various BRB tests and CL load, we found a significant correlation between total, frontal, temporal, and parietal CLs number and pathological scores at BRB tests essentially related to memory, attention, and IPS, as shown in Table 2.

**Table 2:** Correlation between single BRB test score and CLs number

Univariate analysis				
BRB test	Frontal CLs (p)	Temporal CLs (p)	Parietal CLs (p)	Total CLs (p)
SRT‐LTS	0.04	0.008	0.26	0.02
SRT‐CLTR	0.001	<0.001	0.08	<0.001
SPART	0.32	0.33	0.31	0.31
SDMT	0.13	0.13	0.43	0.14
PASAT 3	0.003	0.01	0.03	0.004
PASAT 2	0.29	0.15	0.49	0.26
SRT‐D	0.001	<0.001	0.08	<0.001
SPART‐D	0.001	<0.001	0.08	<0.001
WLG	0.09	0.11	0.27	0.11
STROOP TEST	0.41	0.11	0.18	0.22

BRB, Brief Repeatable Battery; CLs, cortical lesions; SRT‐LTS, Selective Reminding Test–Long‐term Storage; SRT‐CLTR, Selective Reminding Test–Consistent Long‐term Retrieval; SPART, Spatial Recall Test; SDMT, Symbol Digit Modalities Test; PASAT, Paced Auditory Serial Addition Test at 3 and 2 seconds; SRT‐D, Selective Reminding Test–Delayed Recall; SPART‐D, Spatial Recall Test–Delayed; WLG, Word List Generation.

The multivariate (logistic regression) analysis assessing the relative contributions of main demographic, clinical, and MRI variables in predicting DCI revealed only age as independent predictive factor (*B* = 0.147, OR = 1.159, CI 95% 1.059–1.269, *p* < .001), while MCI was correlated also with total CLs number (*B* = 0.388, OR = 1.474, CI 95% 0.964–2.255, *p* = .04), in addition to age (*B* = 0.059, OR = 1.061, CI 95% 1.004–1.122, *p* = .03). Other significant correlations about single BRB tests are shown in Table 3.

**Table 3:** Logistic regression analysis including demographic, clinical, and MRI variables significantly correlated with DCI, MCI, or impairment in single BRB tests

Multivariate analysis					
Dependent variable	Covariate	B	OR	CI 95%	*p*‐value
DCI	Age	0.147	1.159	1.059–1.269	<.001
MCI	Age	0.059	1.061	1.004–1.122	.03
	Total CLs	0.388	1.474	0.964–2.255	.04
PASAT 3	Total CLs	0.107	1.113	0.997–1.242	.04
WLG	EDSS	1.096	2.993	1.004–8.921	.04
STROOP TEST	Age	0.077	1.080	1.003–1.162	.04
	Total CLs	0.596	1.815	1.049–3.142	.02
	EDSS	0.614	1.848	1.087–3.140	.02

DCI, definite cognitive impairment; MCI, mild cognitive impairment; BRB, Brief Repeatable Battery; B, beta coefficient, OR, odds ratio, CI, confidence interval, CLs, cortical lesions; PASAT 3, Paced Auditory Serial Addition Test at 3 seconds; WLG, Word List Generation; EDSS, Expanded Disability Status Scale.

The multivariate analysis conducted in the RRMS subgroup (*n* = 47) demonstrated a significant correlation between total CLs number and SDMT (*p* = .006). Even total T2/FLAIR lesions number was correlated only with SDMT (*p* = .001). As for the entire population, even this subgroup analysis revealed only age as independent predictor of DCI (*p* = .007); age was also correlated with SDMT (*p* < .001).

## 4 DISCUSSION

In our study, CLs were mainly located in the frontal lobe (48.3%), followed by the temporal (33.1%) and the parietal (18.0%) ones; we found only a single occipital lesion (0.6%) in one patient. Moreover, CLs were more common in the progressive forms (*p* = .03). These results are consistent with the literature. In fact, the development of CLs has been reported more frequently in the temporal and frontal lobes, while the occipital lobe is less affected (Calabrese, Favaretto, Martini, & Gallo, 2013). With regard to CLs distribution in different disease subtypes, one recent study reported that in CIS and early RRMS patients, the most affected areas were the fronto‐temporal regions, while CLs were more widespread in late RRMS and SPMS (Calabrese et al., 2015).

There are many evidences supporting a relationship between CLs burden and CI in patients with MS. Most studies, evaluating CLs number and/or volume with different MRI techniques, found a significant correlation between cortical involvement and CI both in RR and progressive patients (Calabrese et al., 2009; Damasceno et al., 2015; Mike et al., 2011; Nelson et al., 2011; Roosendaal et al., 2009), even though there is not universal agreement. In fact, in a 3D‐DIR study on 91 patients with MS or CIS, Papadopoulou et al. (2013) revealed WM lesions volume as a more significant predictor of neuropsychological outcomes than CLs volume, even if these different results from previous works could be explained by differences between the study populations. Besides, in a recent ultra‐high‐field (7.0 T) MRI study, Nielsen et al. found that both WM lesion volume and subpial and leukocortical CLs were strongly associated with CI in MS (Nielsen et al., 2013).

In our study, the presence of CLs was more frequent in patients with CI, even though without statistical significance. However, we found a significant correlation between total, frontal, temporal, and parietal CLs number and pathological performances at BRB tests mainly related to memory, attention, and the most affected cognitive domains in MS. The multivariate analysis confirmed the correlation between total CLs number and the presence of MCI or pathologic score at PASAT 3 and ST. The logistic regression analysis conducted on the RRMS subgroup found results similar to that conducted in all sample, with a significant correlation between total CLs and SDMT, a test which explores mainly IPS, as PASAT 3.

In the interpretation of these results, we have to consider several limitations presented by our study. First, the size of our sample was probably too limited and the “matching” of the two groups too incomplete to allow definitive conclusions. It is likely, in fact, that the lack of a statistically significant correlation between CLs and CI is mainly due to this reason. We had also to consider the lack of a control group well matched to the patients. Secondly, the MRI technique we employed, that is 3D‐DIR, is far from perfect in detecting total CLs burden. A histopathological MRI study describing the postmortem verification of MS CLs detection with 3D‐DIR reported a high sensitivity for mixed GM‐WM lesions (83%), but only 18% for intracortical lesions (Seewann et al., 2012). In addition to that, we have to consider that subpial CLs, which are also associated with physical and cognitive dysfunction in patients with MS, are poorly detected by conventional MRI and even DIR (Geurts et al., 2011, 2012). However, other comparative neuropathological and imaging studies demonstrated that the number of CLs detected at MRI correlates well with their total number revealed by histology, concluding that “the tip of the iceberg detected by MRI and its bulk differ only in size.” (Calabrese et al., 2013; Geurts et al., 2012; Seewann et al., 2011) Then, there are some consensus recommendations for MS CLs scoring using DIR MRI (Geurts et al., 2011), while other more accurate techniques, such as “Phase Sensitive Inversion Recovery” (PSIR) or ultra‐high‐field MRI lack of standardization and are less available in clinical practice.

In addition to the previously reported limitations, we measured only CLs number but not volume, even though several studies reported CLs volume as a more accurate predicting factor of CI. However, volumetric measures are usually limited to research setting, while CLs count can be easily evaluated. Then, the regional CLs distribution we adopted (i.e., cerebral lobes) is quite rough, as it does not consider functional pathways. Finally, we have to consider that CLs burden is only one factor of the complex biological interplay between WM disease and GM disease which likely sustains CI. A lot of previous MRI studies reported correlation between CI and various neuroimaging markers, including WM lesion load and CLs, whole‐brain, cerebellar, deep GM, callosal and cortical atrophy, and normal appearing WM damage (Daams et al., 2016).

In spite of these limitations, our study confirmed the correlation between total CLs number and low performances in several cognitive tests which explore memory, attention, and IPS, as shown in previous studies (Calabrese et al., 2009; Mike et al., 2011; Nelson et al., 2011; Roosendaal et al., 2009). Moreover, we found this correlation also with CLs number detected in single cerebral lobes, with the exception of the occipital one, where we found only one lesion. In our knowledge, there are only sporadic reports in the literature of a direct correlation between regional CLs number and deficit in specific neuropsychological tests, such as one study describing the association between hippocampal lesion number and visuospatial memory (Roosendaal et al., 2009). Besides, some previous studies described the relationship between regional GM atrophy and cognition (Morgen et al., 2006; Nocentini et al., 2014); another study, instead, reported a correlation between frontal and parietal WM lesions volume and impairment on neuropsychological tests evaluating sustained attention, processing speed, and verbal memory (Sperling et al., 2001). Also, our data reported an association between single BRB tests and CLs number detected in various cerebral lobes, according to the aforementioned studies. This lack of anatomic specificity could be explained by the involvement of different cognitive functions associated with several cortical areas during the performance of a single test (Nocentini et al., 2014).

Beyond these considerations about regional CLs measures, and despite the aforementioned limitations, our results highlighted the relevance of total CLs number as a global parameter correlating with CI in patients with MS, a biomarker easy to evaluate also in a clinical practice setting. In fact, while most of the previous studies employed MRI techniques mainly available only in a research setting, we investigated CLs burden with high‐field MRI 3D‐DIR, a technique quite available in several MS centers. In addition to that, as we had already pointed out, detection of CLs number is easier to achieve than volumetric measures.

This correlation, obviously, has relevant clinical implications. In fact, detection of a considerable CLs load ought to recommend a specific neuropsychological assessment, often missed in a routine evaluation. Indeed, an early diagnosis of CI is fundamental, because of its potential dramatic impact on the quality of life and functioning of patients with MS. The monitoring of CLs burden can also be useful to monitor the potential neuroprotective effects of disease‐modifying drugs.

However, large MRI longitudinal studies are needed to explore in detail these implications. We also need to develop more sensitive MRI technologies to increase our capacity to investigate cortical pathology in MS and its complex interaction with GM and WM damage in the determination of MS‐related CI.

## CONFLICT OF INTERESTS

E. Curti has served on scientific advisory boards for Merck Serono and has received funding for travel from Biogen, Merck Serono, Novartis, Sanofi Genzyme, and Roche. F. Granella has received research grants for his Institution from Biogen and Sanofi Genzyme; has served on scientific advisory boards for Biogen, Novartis, Sanofi Genzyme, and Merck Serono; and has received funding for travel from Biogen, Merck Serono, and Sanofi Genzyme; S. Graziuso, E. Tsantes, and G. Crisi have nothing to disclose.

## References

[brb3955-bib-0001] Amato, M. P. , Portaccio, E. , Goretti, B. , Zipoli, V. , Ricchiuti, L. , De Caro, M. F. , … Trojano, M. (2006). The Rao's Brief Repeatable Battery and Stroop Test: Normative values with age, education and gender corrections in an Italian population. Multiple Sclerosis, 12, 787–793. https://doi.org/10.1177/1352458506070933 17263008 10.1177/1352458506070933

[brb3955-bib-0002] Amato, M. P. , Zipoli, V. , & Portaccio, E. (2006). Multiple sclerosis‐related cognitive changes: A review of cross‐sectional and longitudinal studies. Journal of the Neurological Sciences, 245, 41–46. https://doi.org/10.1016/j.jns.2005.08.019 16643953 10.1016/j.jns.2005.08.019

[brb3955-bib-0003] Calabrese, M. , Agosta, F. , Rinaldi, F. , Mattisi, I. , Grossi, P. , Favaretto, A. , … Filippi, M. (2009). Cortical lesions and atrophy associated with cognitive impairment in relapsing‐ remitting multiple sclerosis. Archives of Neurology, 66, 1144–1150. 19752305 10.1001/archneurol.2009.174

[brb3955-bib-0004] Calabrese, M. , De Stefano, N. , Atzori, M. , Bernardi, V. , Mattisi, I. , Barachino, L. , … Gallo, P. (2007). Detection of cortical inflammatory lesions by double inversion recovery magnetic resonance imaging in patients with multiple sclerosis. Archives of Neurology, 64, 1416–1422. https://doi.org/10.1001/archneur.64.10.1416 17923625 10.1001/archneur.64.10.1416

[brb3955-bib-0005] Calabrese, M. , Favaretto, A. , Martini, V. , & Gallo, P. (2013). Grey matter lesions in MS: From histology to clinical implications. Prion, 7, 20–27. https://doi.org/10.4161/pri.22580 23093801 10.4161/pri.22580 PMC3609046

[brb3955-bib-0006] Calabrese, M. , Filippi, M. , Rovaris, M. , Mattisi, I. , Bernardi, V. , Atzori, M. , … Gallo, P. (2008). Morphology and evolution of cortical lesions in multiple sclerosis: A longitudinal MRI study. NeuroImage, 42, 1324–1328. https://doi.org/10.1016/j.neuroimage.2008.06.028 18652903 10.1016/j.neuroimage.2008.06.028

[brb3955-bib-0007] Calabrese, M. , & Gallo, P. (2009). Magnetic resonance evidence of cortical onset of multiple sclerosis. Multiple Sclerosis, 15, 933–941. https://doi.org/10.1177/1352458509106510 19667021 10.1177/1352458509106510

[brb3955-bib-0008] Calabrese, M. , Reynolds, R. , Magliozzi, R. , Castellaro, M. , Morra, A. , Scalfari, A. , … Monaco, S. (2015). Regional distribution and evolution of gray matter damage in different populations of multiple sclerosis patients. PLoS ONE, 10(8), e0135428 https://doi.org/10.1371/journal.pone.0135428 eCollection 2015. 26267665 10.1371/journal.pone.0135428 PMC4534410

[brb3955-bib-0009] Calabrese, M. , Rocca, M. A. , Atzori, M. , Mattisi, I. , Bernardi, V. , Favaretto, A. , … Filippi, M. (2009). Cortical lesions in primary progressive multiple sclerosis: A 2‐year longitudinal MR study. Neurology, 72, 1330–1336. https://doi.org/10.1212/WNL.0b013e3181a0fee5 19365054 10.1212/WNL.0b013e3181a0fee5

[brb3955-bib-0010] Chiaravalloti, N. D. , & DeLuca, J. (2008). Cognitive impairment in multiple sclerosis. Lancet Neurology, 7, 1139–1151. https://doi.org/10.1016/S1474-4422(08)70259-X 19007738 10.1016/S1474-4422(08)70259-X

[brb3955-bib-0011] Daams, M. , Steenwijk, M. D. , Schoonheim, M. M. , Wattjes, M. P. , Balk, L. J. , Tewarie, P. K. , … Barkhof, F. (2016). Multi‐parametric structural magnetic resonance imaging in relation to cognitive dysfunction in long‐standing multiple sclerosis. Multiple Sclerosis, 22, 608–619. https://doi.org/10.1177/1352458515596598 26209593 10.1177/1352458515596598

[brb3955-bib-0012] Damasceno, A. , Damasceno, B. P. , & Cendes, F. (2015). Subclinical MRI disease activity influences cognitive performance in MS patients. Multiple Sclerosis and Related Disorders, 4, 137–143. https://doi.org/10.1016/j.msard.2015.01.006 25787189 10.1016/j.msard.2015.01.006

[brb3955-bib-0013] De Stefano, N. , Matthews, P. M. , Filippi, M. , Agosta, F. , De Luca, M. , Bartolozzi, M. L. , … Smith, S. M. (2003). Evidence of early cortical atrophy in MS: Relevance to white matter changes and disability. Neurology, 60, 1157–1162. https://doi.org/10.1212/01.WNL.0000055926.69643.03 12682324 10.1212/01.wnl.0000055926.69643.03

[brb3955-bib-0014] Geurts, J. J. , Calabrese, M. , Fisher, E. , & Rudick, R. A. (2012). Measurement and clinical effect of grey matter pathology in multiple sclerosis. Lancet Neurology, 11, 1082–1092. https://doi.org/10.1016/S1474-4422(12)70230-2 23153407 10.1016/S1474-4422(12)70230-2

[brb3955-bib-0015] Geurts, J. J. , Pouwels, P. J. , Uitdehaag, B. M. , Polman, C. H. , Barkhof, F. , & Castelijns, J. A. (2005). Intracortical lesions in multiple sclerosis: Improved detection with 3D double inversion‐recovery MR imaging. Radiology, 236, 254–260. https://doi.org/10.1148/radiol.2361040450 15987979 10.1148/radiol.2361040450

[brb3955-bib-0016] Geurts, J. J. , Roosendaal, S. D. , Calabrese, M. , Ciccarelli, O. , Agosta, F. , Chard, D. T. , … MAGNIMS Study Group . (2011). Consensus recommendations for MS cortical lesion scoring using double inversion recovery MRI. Neurology, 76, 418–424. https://doi.org/10.1212/WNL.0b013e31820a0cc4 21209373 10.1212/WNL.0b013e31820a0cc4

[brb3955-bib-0017] Giorgio, A. , Stromillo, M. L. , Rossi, F. , Battaglini, M. , Hakiki, B. , Portaccio, E. , … De Stefano, N. (2011). Cortical lesions in radiologically isolated syndrome. Neurology, 77, 1896–1899. https://doi.org/10.1212/WNL.0b013e318238ee9b 22076541 10.1212/WNL.0b013e318238ee9b

[brb3955-bib-0018] Hautzinger, M. (1991). [The beck depression inventory in clinical practice]. Nervenarzt, 62, 689–696. German. 1770969

[brb3955-bib-0019] Jongen, P. J. , Ter Horst, A. T. , & Brands, A. M. (2012). Cognitive impairment in multiple sclerosis. Minerva Medica, 103, 73–96. 22513513

[brb3955-bib-0020] Kutzelnigg, A. , Lucchinetti, C. F. , Stadelmann, C. , Brück, W. , Rauschka, H. , Bergmann, M. , … Lassmann, H. (2005). Cortical demyelination and diffuse white matter injury in multiple sclerosis. Brain, 128, 2705–2712. https://doi.org/10.1093/brain/awh641 16230320 10.1093/brain/awh641

[brb3955-bib-0021] Lovera, J. , & Kovner, B. (2012). Cognitive Impairment in Multiple Sclerosis. Current Neurology and Neuroscience Reports, 12, 618–627. https://doi.org/10.1007/s11910-012-0294-3 22791241 10.1007/s11910-012-0294-3 PMC4581520

[brb3955-bib-0022] Lucchinetti, C. F. , Popescu, B. F. , Bunyan, R. F. , Moll, N. M. , Roemer, S. F. , Lassmann, H. , … Ransohoff, R. M. (2011). Inflammatory cortical demyelination in early multiple sclerosis. New England Journal of Medicine, 365, 2188–2197. https://doi.org/10.1056/NEJMoa1100648 22150037 10.1056/NEJMoa1100648 PMC3282172

[brb3955-bib-0023] Mike, A. , Glanz, B. I. , Hildenbrand, P. , Meier, D. , Bolden, K. , Liguori, M. , … Guttmann, C. R. (2011). Identification and clinical impact of multiple sclerosis cortical lesions as assessed by routine 3T MR imaging. AJNR. American Journal of Neuroradiology, 32, 515–521. https://doi.org/10.3174/ajnr.A2340 21310857 10.3174/ajnr.A2340 PMC8013100

[brb3955-bib-0024] Morgen, K. , Sammer, G. , Courtney, S. M. , Wolters, T. , Melchior, H. , Blecker, C. R. , … Vaitl, D. (2006). Evidence for a direct association between cortical atrophy and cognitive impairment in relapsing‐remitting MS. NeuroImage, 30, 891–898. https://doi.org/10.1016/j.neuroimage.2005.10.032 16360321 10.1016/j.neuroimage.2005.10.032

[brb3955-bib-0025] Nelson, F. , Datta, S. , Garcia, N. , Rozario, N. L. , Perez, F. , Cutter, G. , … Wolinsky, J. S. (2011). Intracortical lesions by 3T magnetic resonance imaging and correlation with cognitive impairment in multiple sclerosis. Multiple Sclerosis, 17, 1122–1129. https://doi.org/10.1177/1352458511405561 21543552 10.1177/1352458511405561 PMC3151473

[brb3955-bib-0026] Nielsen, A. S. , Kinkel, R. P. , Madigan, N. , Tinelli, E. , Benner, T. , & Mainero, C. (2013). Contribution of cortical lesion subtypes at 7T MRI to physical and cognitive performance in MS. Neurology, 81, 641–649. https://doi.org/10.1212/WNL.0b013e3182a08ce8 23864311 10.1212/WNL.0b013e3182a08ce8 PMC3775688

[brb3955-bib-0027] Nocentini, U. , Bozzali, M. , Spanò, B. , Cercignani, M. , Serra, L. , Basile, B. , … De Luca, J. (2014). Exploration of the relationships between regional grey matter atrophy and cognition in multiple sclerosis. Brain Imaging and Behavior, 8, 378–386. https://doi.org/10.1007/s11682-012-9170-7 22584774 10.1007/s11682-012-9170-7

[brb3955-bib-0028] Papadopoulou, A. , Müller‐Lenke, N. , Naegelin, Y. , Kalt, G. , Bendfeldt, K. , Kuster, P. , … Penner, I. K. (2013). Contribution of cortical and white matter lesions to cognitive impairment in multiple sclerosis. Multiple Sclerosis, 19, 1290–1296. https://doi.org/10.1177/1352458513475490 23459568 10.1177/1352458513475490

[brb3955-bib-0029] Pirko, I. , Lucchinetti, C. F. , Sriram, S. , & Bakshi, R. (2007). Gray matter involvement in multiple sclerosis. Neurology, 68, 634–642. https://doi.org/10.1212/01.wnl.0000250267.85698.7a 17325269 10.1212/01.wnl.0000250267.85698.7a

[brb3955-bib-0030] Popescu, B. F. , & Lucchinetti, C. F. (2012). Meningeal and cortical grey matter pathology in multiple sclerosis. BMC Neurology, 12, 11 https://doi.org/10.1186/1471-2377-12-11 22397318 10.1186/1471-2377-12-11 PMC3315403

[brb3955-bib-0031] Roosendaal, S. D. , Moraal, B. , Pouwels, P. J. , Vrenken, H. , Castelijns, J. A. , Barkhof, F. , & Geurts, J. J. (2009). Accumulation of cortical lesions in MS: Relation with cognitive impairment. Multiple Sclerosis, 15, 708–714. https://doi.org/10.1177/1352458509102907 19435749 10.1177/1352458509102907

[brb3955-bib-0032] Seewann, A. , Kooi, E. J. , Roosendaal, S. D. , Pouwels, P. J. , Wattjes, M. P. , van der Valk, P. , … Geurts, J. J. (2012). Postmortem verification of MS cortical lesion detection with 3D DIR. Neurology, 78, 302–308. https://doi.org/10.1212/WNL.0b013e31824528a0 22218278 10.1212/WNL.0b013e31824528a0

[brb3955-bib-0033] Seewann, A. , Vrenken, H. , Kooi, E. J. , van der Valk, P. , Knol, D. L. , Polman, C. H. , … Geurts, J. J. (2011). Imaging the tip of the iceberg: Visualization of cortical lesions in multiple sclerosis. Multiple Sclerosis, 17, 1202–1210. https://doi.org/10.1177/1352458511406575 21561955 10.1177/1352458511406575

[brb3955-bib-0034] Sperling, R. A. , Guttmann, C. R. , Hohol, M. J. , Warfield, S. K. , Jakab, M. , Parente, M. , … Weiner, H. L. (2001). Regional magnetic resonance imaging lesion burden and cognitive function in multiple sclerosis: A longitudinal study. Archives of Neurology, 58, 115–121. 11176944 10.1001/archneur.58.1.115

[brb3955-bib-0035] Turetschek, K. , Wunderbaldinger, P. , Bankier, A. A. , Zontsich, T. , Graf, O. , Mallek, R. , & Hittmair, K. (1998). Double inversion recovery imaging of the brain: Initial experience and comparison with fluid attenuated inversion recovery imaging. Magnetic Resonance Imaging, 16, 127–135. https://doi.org/10.1016/S0730-725X(97)00254-3 9508269 10.1016/s0730-725x(97)00254-3

